# Supplementary material for: Fermiology of Chiral Cadmium Diarsenide CdAs2, a Candidate for Hosting Kramers–Weyl Fermions
Source: J Phys Chem Lett. 2023 Mar 23;14(13):3120–5. doi: 10.1021/acs.jpclett.3c00005 (PMC10084463; doi:10.1021/acs.jpclett.3c00005)
Supplement: Supplementary file 1 — jz3c00005_si_001.pdf [file jz3c00005_si_001.pdf]

THE JOURNAL OF PHYSICAL CHEMISTRY LETTERS
A JOURNAL OF THE AMERICAN CHEMICAL SOCIETY

pubs.acs.org/JPCL

Letter

Fermiology of Chiral Cadmium Diarsenide $CdAs_2$, a Candidate for Hosting Kramers–Weyl Fermions

Federico Mazzola,[¶] Yanxue Zhang,[¶] Natalia Olszowska,[¶] Marcin Rosmus,[¶] Gianluca D'Olimpio, Marian Cosmin Istrate, Grazia Giuseppina Politano, Ivana Vobornik, Raman Sankar, Corneliu Ghica, Junfeng Gao,* and Antonio Politano*

Cite This: J. Phys. Chem. Lett. 2023, 14, 3120–3125

Read Online

ACCESS | Metrics & More | Article Recommendations | SI Supporting Information

ABSTRACT: Nonmagnetic chiral crystals are a new class of systems hosting Kramers–Weyl Fermions, arising from the combination of structural chirality, spin–orbit coupling (SOC), and time-reversal symmetry. These materials exhibit nontrivial Fermi surfaces with SOC-induced Chern gaps over a wide energy range, leading to exotic transport and optical properties. In this study, we investigate the electronic structure and transport properties of $CdAs_2$, a newly reported chiral material. We use synchrotron-based angle-resolved photoelectron spectroscopy (ARPES) and density functional theory (DFT) to determine the Fermiology of the (110)-terminated $CdAs_2$ crystal. Our results, together with complementary magnetotransport measurements, suggest that $CdAs_2$ is a promising candidate for novel topological properties protected by the structural chirality of the system. Our work sheds light on the details of the Fermi surface and topology for this chiral quantum material, providing useful information for engineering novel spintronic and optical devices based on quantized chiral charges, negative longitudinal magnetoresistance, and nontrivial Chern numbers.

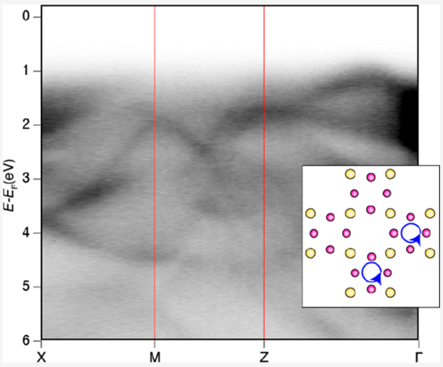

Crystal symmetries play a pivotal role in determining the electronic properties of various quantum systems, and their study has garnered substantial interest for both fundamental research and technological applications.[1−9] Crystals exhibiting well-defined handedness, due to the breaking of inversion, mirror, or any other roto-inversion symmetries, are referred to as chiral crystals.[10−12] Even in their nonmagnetic state, these chiral crystals show universal topological electronic properties due to their spin–orbit coupling and crystalline chirality, resulting in the presence of Kramers–Weyl Fermions in their spectrum.[13,14] These Fermions are pinned to Kramers degenerate points, leading to the appearance of topological gaps, which are significantly larger than those observed in Weyl semimetals.[13] Within such gaps, ubiquitous topological properties, such as quantized chiral charges,[15] negative longitudinal magnetoresistance,[16] and nontrivial Chern numbers[17] can arise, opening up exciting avenues for engineering exotic transport phenomena and applications. Furthermore, Kramers–Weyl Fermions differ from conventional Weyl Fermions as they occur at time-reversal invariant points in momentum space. SOC, structural chirality, and time-reversal symmetry combine to produce these unique properties, which can also enable additional exotic phenomena such as magneto-chiral dichroism,[18,19] large optical activity,[20,21] and even the emergence of skyrmions with the lifting of time-reversal symmetry.[22,23] As such, understanding the electronic structure of these compounds is of paramount importance, especially given the evident potential for technological applications in spintronics and optics.[11]

In this study, we investigate the electronic structure of the newly reported[24,25] chiral material $CdAs_2$ using synchrotron-based angle-resolved photoelectron spectroscopy (ARPES), density functional theory (DFT), and transport experiments, shedding light on the details of the Fermi surface and topology for this chiral quantum material. Our findings suggest that $CdAs_2$ is a promising candidate for enabling novel topological properties protected by its structural chirality, offering useful information for the development of disruptive spintronic and optical devices based on quantized chiral charges, negative longitudinal magnetoresistance, and nontrivial Chern numbers.

$CdAs_2$ exhibits trigonal symmetry belonging to the space group n. 98, $I4_122$(see Figure 1). The lattice parameters $a = b = 8.152$ Å and $c = 4.771$ Å were determined from our X-ray diffraction (XRD) measurements (Figure 2c). The crystal structure reflects the chirality of the system, with atoms forming spiral chains of covalent As–As and Cd–As bonds

Received: January 2, 2023
Accepted: March 20, 2023

© XXXX American Chemical Society

https://doi.org/10.1021/acs.jpclett.3c00005
J. Phys. Chem. Lett. 2023, 14, 3120–3125

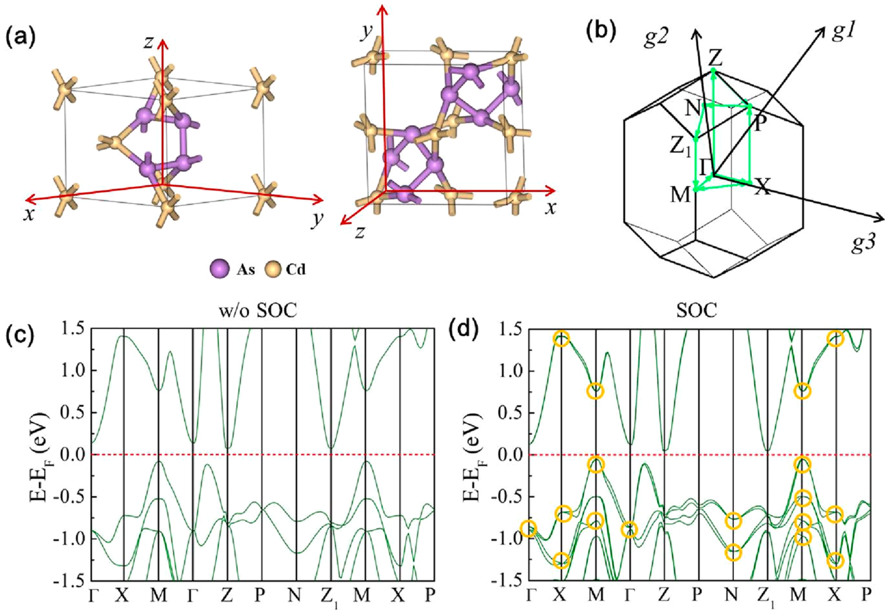

Figure 1. Crystal structure and electronic band structure of bulk $CdAs_2$. (a) Optimized primitive (left) and conventional (right) unit cells of $CdAs_2$. (b) First Brillouin zone of the primitive cell. (c) Calculated electronic band structure without SOC effects. (d) Calculated electronic band structure including SOC effects. The Fermi level is set to zero and marked by a horizontal red dashed line. Cd and As atoms are represented by yellow and purple balls, respectively. The Kramers–Weyl nodes (d) are marked by yellow circles.

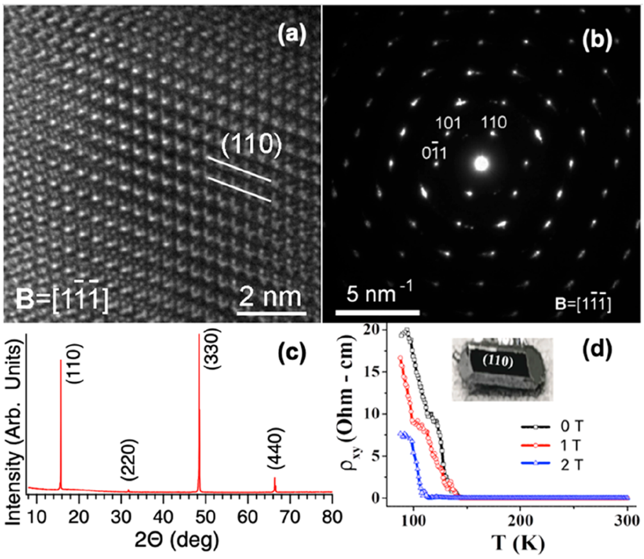

Figure 2. Characterization of the $CdAs_2$ single crystal. (a) High-resolution transmission electron microscopy (HR-TEM) image showing the crystalline structure of $CdAs_2$. (b) Small-area electron diffraction (SAED) pattern confirming the single crystal nature of $CdAs_2$, although the existence of elongated spots is a fingerprint of a slight mosaicity. (c) X-ray diffraction (XRD) pattern revealing the high quality of the crystal with sharp Bragg peaks. (d) Temperature-dependent magnetoresistance curve of $CdAs_2$ single crystal showing a linear dependence at low temperatures and a saturation behavior at high temperatures.

(Figure 1a), which influence both the optical and electronic properties.

The electronic structure of the bulk, along the high-symmetry directions (for Brillouin zone, BZ, see Figure 1b), shows a small indirect energy gap (~0.13 eV) as displayed in Figure 1c, indicating the semiconducting nature of the bulk crystal. The inclusion of SOC reduces the gap by approximately 20 meV, as shown in Figure 1d, but the semiconducting character is retained. With the inclusion of SOC, the band structure of $CdAs_2$ displays a 2-fold splitting, whereas in its absence, it exhibits a 2-fold spin degeneracy. However, at time-reversal-invariant-momenta, 2-fold spin degeneracy is retained even in the presence of SOC, which is crucial for the emergence of Kramers–Weyl Fermions.[11,12]

We calculated the Wannier charge centers for six time reversal invariant planes: k_1 = (0.0, 0.5), k_2 = (0.0, 0.5), k_3 = (0.0, 0.5) of primitive $CdAs_2$, as shown in Figure S4 in the Supporting Information. The results indicate that the k_2–k_3 plane and the k_1–k_3 plane have a reverse topological number $\mathbb{Z}_2$, i.e., $\mathbb{Z}_2(k_1 = 0) = \mathbb{Z}_2(k_2 = 0.5) = 0$, $\mathbb{Z}_2(k_1 = 0.5) = \mathbb{Z}_2(k_2 = 0.0) = 1$. We hypothesized that this may be attributed to the structural chirality of $CdAs_2$. It is worth noting that the surface electronic structure may differ significantly from the bulk. In this study, we examine four possible surface terminations along the (110) plane: As–S1, As–S2, Cd–S1, and Cd–S2 (refer to Figure S1 in the Supporting Information). Using DFT calculations, we found that the As–S1 and Cd–S1 surfaces are notably more stable than the others, without any observed distortions. Conversely, the As–S2 and Cd–S2 surfaces exhibit reconstruction, where the topmost Cd atoms in Cd–S2 sink into the As-sublayer. This behavior is comparable to a self-passivation mechanism reported in three-dimensional Dirac semimetal Cd_3As_2.[26]

To determine the most realistic configuration for comparison to the experiment, we relaxed the (2 × 1) and (3 × 1) supercells of As–S1, As–S2, Cd–S1, and Cd–S2 terminated (110) surface to simulate their electronic structure (Figure 3). The surface formation energy as a function of the chemical potential of As atoms (μ_{As}) for the four types of surfaces (with a thickness of 17 Å) is illustrated in Figure S2a. The Cd–S1 termination exhibits the lowest surface energy for lower μ_{As} (blue line in Figure S2a in the Supporting Information), following a linear trend. Conversely, the As–S1 termination

https://doi.org/10.1021/acs.jpclett.3c00005
J. Phys. Chem. Lett. 2023, 14, 3120–3125

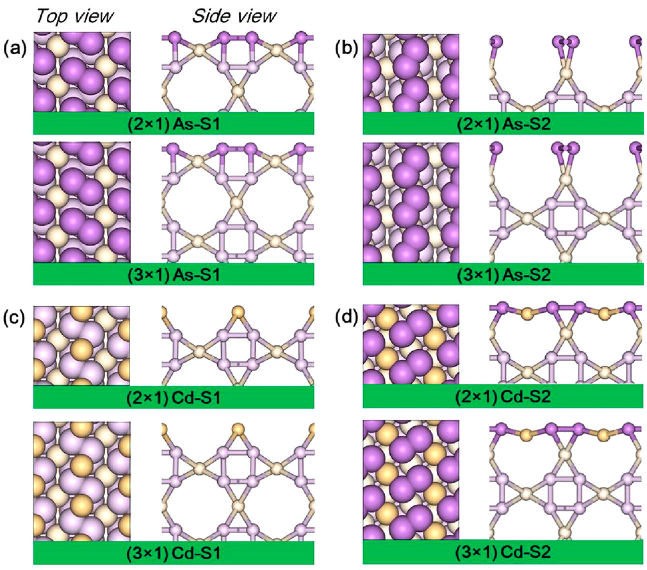

Figure 3. Top (left panel) and side (right panel) view of (2 × 1) (top panel) and (3 × 1) (bottom panel) supercell of (a) As−S1, (b) As−S2, (c) Cd−S1, and (d) Cd−S2, respectively. Lighter colors represent lower atoms for better visualization of surface atoms.

yields the lowest surface energy in a richer As environment. This trend holds for various thicknesses; the surface energy appears unaffected by slab thickness (refer to Figure S2a−d in the Supporting Information for calculations from 17 to 35 Å). Therefore, we focus on the As−S1 and Cd−S1 surfaces, which exhibit the lowest and most favorable surface energy configurations, in our further analysis of (110)-oriented crystals.

$CdAs_2$ single crystals with (110) orientation were analyzed using high-resolution transmission electron microscopy (HR-TEM, Figure 2a,b). The unit cell parameters, determined to be $a = b = 0.795$ nm and $c = 0.467$ nm, were found to be consistent with previous reports[27] and XRD results (Figure 2c). Temperature-dependent magnetoresistance measurements were carried out on the same crystals, revealing a semiconductor-metal transition that is quenched with increasing magnetic field. The data in Figure 2d demonstrate that the semiconducting behavior persists below 150 K and becomes metallic above this threshold.[28] The experimental results support the conclusion that $CdAs_2$ undergoes a transition from semiconductor to metal with increasing temperature.

The electronic structure of $CdAs_2$ was probed using synchrotron-based ARPES. Consistent with the bulk semiconducting nature of the material, the ARPES spectra showed an energy gap separating the valence and conduction bands (Figure 4 and 5). The constant energy maps (Figure 4a−c) revealed a complex Fermiology, particularly for the valence band manifold, with small metallic conduction band pockets comprising the Fermi surface (Figure 4a). The energy-momentum dispersion along high-symmetry directions (Figure 5) indicated the presence of strongly dispersing bands with broad features, suggestive of the three-dimensional nature of the material, which introduces a significant k_z contribution in ARPES measurements.

DFT calculations predicted the presence of metallic in-gap surface states that cross the Fermi level for the most stable As-terminated surface, where the presence of dangling bonds is expected to generate these states. Band structures were calculated for As−S1 (110) and Cd−S1 (110) surfaces with thicknesses of 17−35 Å (Figure S3 in the Supporting Information), revealing that the surface states of As−S1 (110) and Cd−S1 (110) surfaces are metallic and cross the Fermi energy level (Figure S5 in the Supporting Information). The As−S1 (110) surface showed decreasing conduction bands at the Γ point as thickness increased from 17 to 35 Å, while the conduction band at Γ from the topmost As atoms of As−S1 (110) surface decreased from 0.43 to 0.34 eV. The Cd−S1 (110) surface, on the other hand, showed surface states that crossed the Fermi energy level regardless of the slab thickness, indicating that the Cd−S1 surface is more active and less stable than the As−S1 surface. To further investigate the surface state of As−S1 and Cd−S1 (110) surfaces, Wannier90 code[29] and Wannier Tools[30] package were used (Figure S4 in the Supporting Information). A tight-binding model generated by Wannier90 code confirmed that the surface state of As−S1 (110) surface slightly crosses the Fermi energy level in the direction of Γ to X, while the Cd−S1 (110) surface has several surface states crossing the Fermi energy level.

Although the identification of surface states in ARPES spectra was challenging due to their broad and intense spectral features, derivative plots of the bands (Figure 5) facilitated a more detailed comparison between theory and experiment, revealing additional dispersing features in the gap region, potentially attributed to the surface states. The comparison of

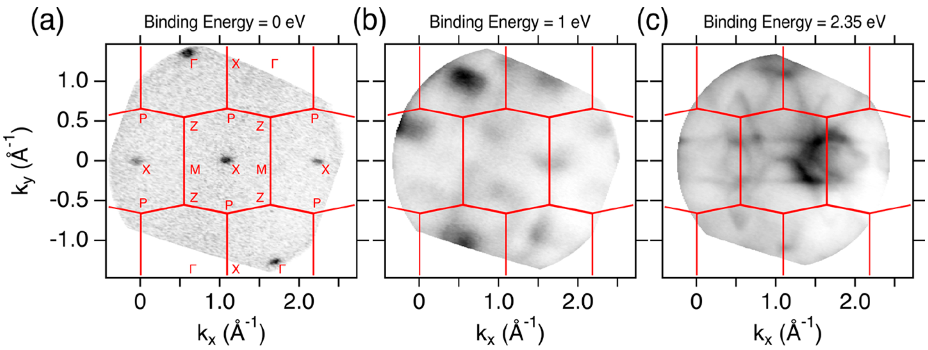

Figure 4. (a) Fermi surface of $CdAs_2$ showing the electron pockets of the conduction band at the Fermi level, covering several Brillouin zones. (b) Constant energy surface at 1 eV and (c) 2.35 eV below the Fermi level illustrating the valence band structure evolution at higher k-values. The measurements were carried out at 40 K using $h\nu = 100$ eV photons in horizontal polarization setups. On the constant energy cuts, the projection of the Wigner−Seitz cells were overlaid and high-symmetry points were marked.

https://doi.org/10.1021/acs.jpclett.3c00005
J. Phys. Chem. Lett. 2023, 14, 3120−3125

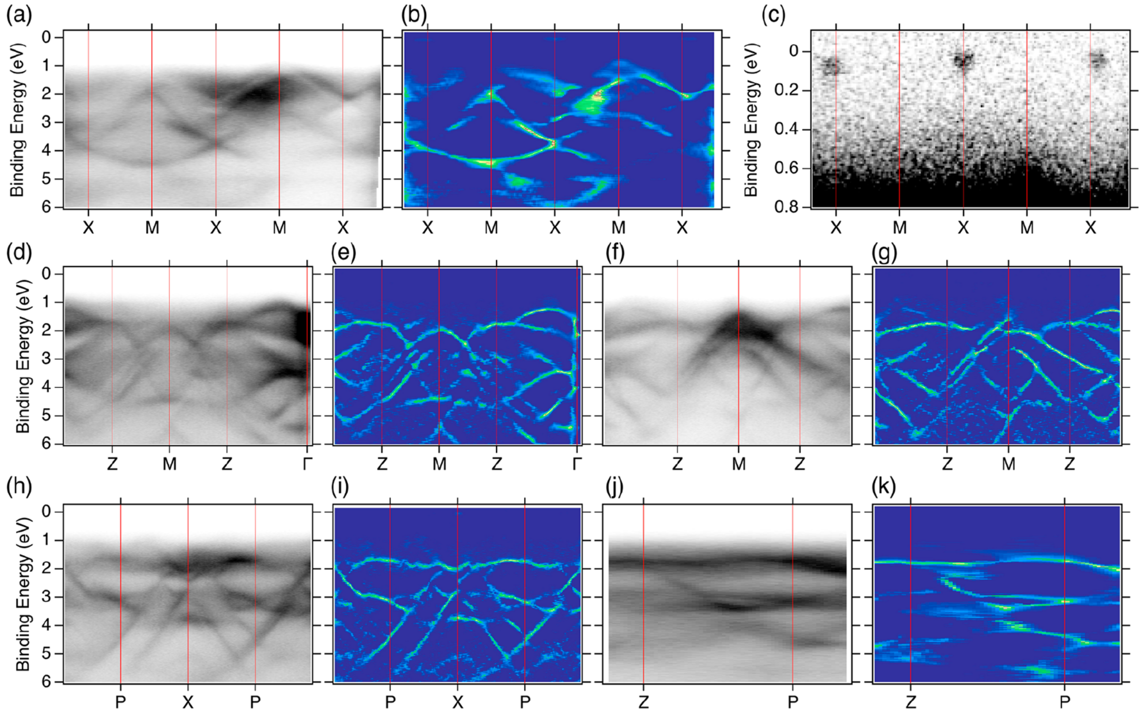

Figure 5. Experimental band structure of bulk $CdAs_2$ along various high-symmetry directions. (a) ARPES repeated along the X−M−X path, showing a large view of the valence band structure. (b) Second derivative plot to aid the visualization of the states. (c) Zoomed-in view of part a near the Fermi level, highlighting the pockets belonging to the conduction band manifold. (d) ARPES valence band structure measured along the Z−M−Z path (negative k_x values). (e) Corresponding second derivative plot. The Z−M−Z path was also collected at positive k_x values and shown in part f along with the (g) second derivative. The valence band was also measured along the (h, i) P−X−P path and (j, k) Z−P direction.

theoretical band structure in Figure 1d with the experimental results in Figure 5 showed a maximum of the valence band at the M point, where Kramers−Weyl Fermions are imposed by the T symmetry.

The chirality and lack of mirror (inversion) symmetry in $CdAs_2$ are of great significance, as they give rise to topological gaps that are much larger than those found in conventional Weyl semimetals. These gaps are clearly evident in our ARPES experiment and make $CdAs_2$ an ideal platform for studying a range of unique phenomena. Notably, the topological properties of this material enable the existence of Kramers−Weyl Fermions, which have been suggested as promising candidates for developing novel spin-torque devices and quantum solenoids.[31]

In summary, our study has provided a comprehensive investigation of the electronic properties of $CdAs_2$. Our results demonstrate that $CdAs_2$ is a semiconducting chiral material with a small gap that can be overcome by thermal activation, leading to a semiconducting-metal transition. We found that the topological properties of this material are mediated by SOC, although it has a minor effect in reducing the gap size. Furthermore, we showed that the presence of metallic states on the material's surface is crucial in enabling additional metallic states. Although identifying these surface states by ARPES is complicated due to the broadening of the spectra, our experimental results are consistent with our theoretical model.

Our findings have significant implications for the development of optical and spintronic devices based on quantized chiral charges, negative longitudinal magnetoresistance, and nontrivial Chern numbers associated with this chiral quantum material and its Kramers−Weyl Fermions.

■ METHODS

Theory. First-principles calculations were performed by using the Vienna ab initio simulation package (VASP).[32] The exchange-correlation interaction was described using the Perdew−Burke−Ernzerhof (PBE) functional[33] in the generalized gradient approximation (GGA), with core electrons described by the Projector-augmented wave (PAW) technology.[34] A plane-wave basis kinetic energy cutoff of 500 eV and a convergence criterion of 10^{-5} eV were used in the calculations. All configurations were fully relaxed until the force was lower than 0.02 eV/Å, with a k-point sampling of 0.02 1/Å used for structural relaxation.

Crystal Growth. $CdAs_2$ single crystals were grown by the Chemical Vapor Transport (CVT) method, using Cd metal chunks (purity 99.99%), As chunks (purity 99.999%), and I_2 transport agent (purity 99.999% analytical grade) purchased from Alfa Aesar Chemical in a weight ratio of 43:57. The growth process was carried out in a carbon-coated quartz tube, sealed under an Ar gas atmosphere using a glovebox. A horizontal two-zone furnace with programmable temperature and time was used to maintain the furnace hot and cold zones at 600 and 550 °C, respectively, for 2 weeks. The grown crystals were collected, washed in a glovebox to protect them from surface oxidation, and further washed with ethanol to remove any surface contamination from I_2.

TEM. HR-TEM investigation of the grain surface was performed on crystal grains hanging freely in carbon membrane holes with no support underneath. We selected grains tilted to the nearest available zone axis orientation, as shown in the selected area electron diffraction (SAED) patterns in Figure 2b. HR-TEM micrographs were acquired from the thinnest regions at the grain border.

https://doi.org/10.1021/acs.jpclett.3c00005
J. Phys. Chem. Lett. 2023, 14, 3120−3125

ARPES. ARPES measurements were carried out at the National Synchrotron Radiation Centre SOLARIS in Cracow, Poland, using the variable polarization and high-resolution URANOS beamline depicted in Figure S6 in the Supporting Information. Samples were glued with epoxy resin to a sample holder and cleaved in an ultrahigh vacuum by a metal post. The experiment was conducted using a quasiperiodic elliptically polarizing undulator APPLE II type as a photon source. Experimental data were collected by a VGScienta DA30L electron spectrometer, with an energy and angle resolution better than 3 meV and 0.1°, respectively. Data measurements were performed at T = 40 K and for an energy range from 20 to 140 eV. The spot size on the sample was 250 × 250 μm.

Transport Experiments. The temperature-dependent transport properties were investigated using a 4-probe measurement with the magnetic field varying from 0 to 2 T in a Quantum Design (QD) based Physical Property Measurement System (PPMS).

■ ASSOCIATED CONTENT

Supporting Information

The Supporting Information is available free of charge at https://pubs.acs.org/doi/10.1021/acs.jpclett.3c00005.

Further information on the theoretical model and geometry of ARPES experiments (PDF)

■ AUTHOR INFORMATION

Corresponding Authors

Junfeng Gao − *Key Laboratory of Materials Modification by Laser, Ion and Electron Beams, Ministry of Education, School of Physics, Dalian University of Technology, Dalian 116024, China;* orcid.org/0000-0001-5732-9905; Email: gaojf@dlut.edu.cn

Antonio Politano − ~~*National Institute of Materials Physics, 077125 Magurele, Romania*~~; orcid.org/0000-0002-4254-2102; Email: antonio.politano@univaq.it

Authors

Federico Mazzola − *Istituto Officina dei Materiali (IOM)−CNR, Laboratorio TASC, I-34149 Trieste, Italy; Department of Molecular Sciences and Nanosystems, Ca' Foscari University of Venice, I-30172 Venice, Italy*

Yanxue Zhang − *Key Laboratory of Materials Modification by Laser, Ion and Electron Beams, Ministry of Education, School of Physics, Dalian University of Technology, Dalian 116024, China*

Natalia Olszowska − *National Synchrotron Radiation Centre SOLARIS, Jagiellonian University, PL-30392 Kraków, Poland*

Marcin Rosmus − *National Synchrotron Radiation Centre SOLARIS, Jagiellonian University, PL-30392 Kraków, Poland;* orcid.org/0000-0002-4314-9601

Gianluca D'Olimpio − *Department of Physical and Chemical Sciences, University of L'Aquila, I-67100 L'Aquila, AQ, Italy;* orcid.org/0000-0002-6367-3945

Marian Cosmin Istrate − *National Institute of Materials Physics, 077125 Magurele, Romania*

Grazia Giuseppina Politano − *Department of Information Engineering, Infrastructures and Sustainable Energy (DIIES), University "Mediterranea" of Reggio Calabria, I-89122 Reggio Calabria, Italy*

Ivana Vobornik − *Istituto Officina dei Materiali (IOM)−CNR, Laboratorio TASC, I-34149 Trieste, Italy;* orcid.org/0000-0001-9957-3535

Raman Sankar − ~~*Center for Condensed Matter Sciences, National Taiwan University, Taipei 10617, Taiwan*~~; orcid.org/0000-0003-4702-2517

Corneliu Ghica − *National Institute of Materials Physics, 077125 Magurele, Romania;* orcid.org/0000-0001-7110-1544

Complete contact information is available at: https://pubs.acs.org/10.1021/acs.jpclett.3c00005

Author Contributions

¶F.M., Y.Z., N.O., and M.R. contributed equally.

Notes

The authors declare no competing financial interest.

■ ACKNOWLEDGMENTS

This work was supported by the National Natural Science Foundation of China (Grant No. 12074053, 91961204, 12004064), and National Foreign Expert Project (G2022127004L). A.P. thanks CERIC-ERIC for the access to the TEM facility.

■ REFERENCES

(1) Kang, L.; Cao, Z. Y.; Wang, B. Pressure-Induced Electronic Topological Transition and Superconductivity in Topological Insulator $Bi_2Te_{2.1}Se_{0.9}$. *J. Phys. Chem. Lett.* **2022**, *13*, 11521−11527.

(2) Kirby, R. J.; Scholes, G. D.; Schoop, L. M. Square-Net Topological Semimetals: How Spectroscopy Furthers Understanding and Control. *J. Phys. Chem. Lett.* **2022**, *13*, 838−850.

(3) Klimovskikh, I. I.; Estyunin, D. A.; Makarova, T. P.; Tereshchenko, O. E.; Kokh, K. A.; Shikin, A. M. Electronic Structure of Pb Adsorbed Surfaces of Intrinsic Magnetic Topological Insulators. *J. Phys. Chem. Lett.* **2022**, *13*, 6628−6634.

(4) Li, L.; Gunasekaran, S.; Wei, Y.; Nuckolls, C.; Venkataraman, L. Reversed Conductance Decay of 1d Topological Insulators by Tight-Binding Analysis. *J. Phys. Chem. Lett.* **2022**, *13*, 9703−9710.

(5) Li, Y.; Zhang, Y. F.; Deng, J.; Dong, W. H.; Sun, J. T.; Pan, J.; Du, S. Rational Design of Heteroanionic Two-Dimensional Materials with Emerging Topological, Magnetic, and Dielectric Properties. *J. Phys. Chem. Lett.* **2022**, *13*, 3594−3601.

(6) Liang, Y.; Zheng, F.; Zhao, P.; Wang, Q.; Frauenheim, T. Intrinsic Ferroelectric Quantum Spin Hall Insulator in Monolayer Na_3Bi with Surface Trimerization. *J. Phys. Chem. Lett.* **2022**, *13*, 11059−11064.

(7) Xi, M.; Chen, F.; Gong, C.; Tian, S.; Yin, Q.; Qian, T.; Lei, H. Relationship between Antisite Defects, Magnetism, and Band Topology in $MnSb_2Te_4$ Crystals with $T_c \approx 40$ K. *J. Phys. Chem. Lett.* **2022**, *13*, 10897−10904.

(8) Yakovlev, D. S.; Lvov, D. S.; Emelyanova, O. V.; Dzhumaev, P. S.; Shchetinin, I. V.; Skryabina, O. V.; Egorov, S. V.; Ryazanov, V. V.; Golubov, A. A.; Roditchev, D.; Stolyarov, V. S. Physical Vapor Deposition Features of Ultrathin Nanocrystals of $Bi_2(Te_xSe_{1-X})_3$. *J. Phys. Chem. Lett.* **2022**, *13*, 9221−9231.

(9) (a) Zhou, Z.; Peng, K.; Xiao, S.; Wei, Y.; Dai, Q.; Lu, X.; Wang, G.; Zhou, X. Anomalous Thermoelectric Performance in Asymmetric Dirac Semimetal BaAgBi. *J. Phys. Chem. Lett.* **2022**, *13*, 2291−2298. (b) Borca, B.; Michnowicz, T.; Aguilar-Galindo, F.; Pétuya, R.; Pristl, M.; Schendel, V.; Pentegov, I.; Kraft, U.; Klauk, H.; Wahl, P.; et al. Chiral and Catalytic Effects of Site-Specific Molecular Adsorption. *J. Phys. Chem. Lett.* **2023**, *14* (8), 2072−2077.

(10) Sanchez, D. S.; Belopolski, I.; Cochran, T. A.; Xu, X.; Yin, J.-X.; Chang, G.; Xie, W.; Manna, K.; Süß, V.; Huang, C.-Y.; et al. Topological Chiral Crystals with Helicoid-Arc Quantum States. *Nature* **2019**, *567*, 500−505.

(11) Chang, G.; Wieder, B. J.; Schindler, F.; Sanchez, D. S.; Belopolski, I.; Huang, S.-M.; Singh, B.; Wu, D.; Chang, T.-R.; Neupert, T.; Xu, S.-Y.; Lin, H.; Hasan, M. Z. Topological Quantum Properties of Chiral Crystals. *Nat. Mater.* **2018**, *17*, 978−985.

(12) Hasan, M. Z.; Chang, G.; Belopolski, I.; Bian, G.; Xu, S.-Y.; Yin, J.-X. Weyl, Dirac and High-Fold Chiral Fermions in Topological Quantum Matter. *Nat. Rev. Mater.* **2021**, *6*, 784−803.

(13) Tan, W.; Jiang, X.; Li, Y.; Wu, X.; Wang, J.; Huang, B. A Unified Understanding of Diverse Spin Textures of Kramers−Weyl Fermions in Nonmagnetic Chiral Crystals. *Adv. Funct. Mater.* **2022**, *32*, 2208023.

(14) Shekhar, C. Chirality Meets Topology. *Nat. Mater.* **2018**, *17*, 953−954.

(15) Chang, G.; Xu, S.-Y.; Wieder, B. J.; Sanchez, D. S.; Huang, S.-M.; Belopolski, I.; Chang, T.-R.; Zhang, S.; Bansil, A.; Lin, H.; et al. Unconventional Chiral Fermions and Large Topological Fermi Arcs in RhSi. *Phys. Rev. Lett.* **2017**, *119*, 206401.

(16) Arnold, F.; Shekhar, C.; Wu, S.-C.; Sun, Y.; Dos Reis, R. D.; Kumar, N.; Naumann, M.; Ajeesh, M. O.; Schmidt, M.; Grushin, A. G.; et al. Negative Magnetoresistance without Well-Defined Chirality in the Weyl Semimetal TaP. *Nat. Commun.* **2016**, *7*, 1−7.

(17) Tang, P.; Zhou, Q.; Zhang, S.-C. Multiple Types of Topological Fermions in Transition Metal Silicides. *Phys. Rev. Lett.* **2017**, *119*, 206402.

(18) Rikken, G.; Raupach, E. Observation of Magneto-Chiral Dichroism. *Nature* **1997**, *390*, 493−494.

(19) Train, C.; Gheorghe, R.; Krstic, V.; Chamoreau, L.-M.; Ovanesyan, N. S.; Rikken, G. L.; Gruselle, M.; Verdaguer, M. Strong Magneto-Chiral Dichroism in Enantiopure Chiral Ferromagnets. *Nat. Mater.* **2008**, *7*, 729−734.

(20) Hannam, K.; Powell, D. A.; Shadrivov, I. V.; Kivshar, Y. S. Broadband Chiral Metamaterials with Large Optical Activity. *Phys. Rev. B* **2014**, *89*, 125105.

(21) Zhao, R.; Zhang, L.; Zhou, J.; Koschny, T.; Soukoulis, C. Conjugated Gammadion Chiral Metamaterial with Uniaxial Optical Activity and Negative Refractive Index. *Phys. Rev. B* **2011**, *83*, 035105.

(22) Tokunaga, Y.; Yu, X.; White, J.; Rønnow, H. M.; Morikawa, D.; Taguchi, Y.; Tokura, Y. A New Class of Chiral Materials Hosting Magnetic Skyrmions Beyond Room Temperature. *Nat. Commun.* **2015**, *6*, 1−7.

(23) Jena, J.; Göbel, B.; Ma, T.; Kumar, V.; Saha, R.; Mertig, I.; Felser, C.; Parkin, S. S. Elliptical Bloch Skyrmion Chiral Twins in an Antiskyrmion System. *Nat. Commun.* **2020**, *11*, 1−9.

(24) Żdanowicz, E.; Wojciechowski, W.; Misiewicz, J.; Lisunov, K. Negative Magnetoresistance for Different Orientations of N-Type $Cdas_2$ in the Variable-Range Hopping Regime. *Mater. Sci. Eng., B* **1994**, *26*, 19−24.

(25) Oubraham, A.; Biskupski, G.; Zdanowicz, E. Negative Magnetoresistance of N-Type Compensated Cadmium Arsenide ($CdSs_2$) in the Temperature Range 11 K-4.2 K. *Solid State Commun.* **1991**, *77*, 351−354.

(26) Gao, J.; Cupolillo, A.; Nappini, S.; Bondino, F.; Edla, R.; Fabio, V.; Sankar, R.; Zhang, Y. W.; Chiarello, G.; Politano, A. Surface Reconstruction, Oxidation Mechanism, and Stability of Cd_3as_2. *Adv. Funct. Mater.* **2019**, *29*, 1900965.

(27) Červinka, L.; Hrubý, A. The Crystal Structure of $CdSs_2$. *Acta Crystallogr. B* **1970**, *26*, 457−458.

(28) Sales, B.; Jones, E.; Chakoumakos, B.; Fernandez-Baca, J.; Harmon, H.; Sharp, J.; Volckmann, E. Magnetic, Transport, and Structural Properties of $Fe_{1-X}Ir_xSi$. *Phys. Rev. B* **1994**, *50*, 8207.

(29) Pizzi, G.; Vitale, V.; Arita, R.; Blugel, S.; Freimuth, F.; Geranton, G.; Gibertini, M.; Gresch, D.; Johnson, C.; Koretsune, T.; Ibanez-Azpiroz, J.; Lee, H.; Lihm, J. M.; Marchand, D.; Marrazzo, A.; Mokrousov, Y.; Mustafa, J. I.; Nohara, Y.; Nomura, Y.; Paulatto, L.; Ponce, S.; Ponweiser, T.; Qiao, J.; Thole, F.; Tsirkin, S. S.; Wierzbowska, M.; Marzari, N.; Vanderbilt, D.; Souza, I.; Mostofi, A. A.; Yates, J. R. Wannier90 as a Community Code: New Features and Applications. *J. Phys.: Condens. Matter* **2020**, *32*, 165902.

(30) Wu, Q.; Zhang, S.; Song, H.-F.; Troyer, M.; Soluyanov, A. A. Wanniertools: An Open-Source Software Package for Novel Topological Materials. *Comput. Phys. Commun.* **2018**, *224*, 405−416.

(31) He, W.-Y.; Xu, X. Y.; Law, K. T. Kramers Weyl Semimetals as Quantum Solenoids and Their Applications in Spin-Orbit Torque Devices. *Commun. Phys.* **2021**, *4*, 1−8.

(32) Hafner, J. Ab-Initio Simulations of Materials Using Vasp: Density-Functional Theory and Beyond. *J. Comput. Chem.* **2008**, *29*, 2044−2078.

(33) Perdew, J. P.; Burke, K.; Ernzerhof, M. Generalized Gradient Approximation Made Simple. *Phys. Rev. Lett.* **1996**, *77*, 3865−3868.

(34) Kresse, G.; Joubert, D. From Ultrasoft Pseudopotentials to the Projector Augmented-Wave Method. *Phys. Rev. B* **1999**, *59*, 1758−1775.
